# Assessment of self-care practice amongst patients with type II diabetes attending Adama Hospital Medical College, Ethiopia

**DOI:** 10.1186/s12902-022-01049-9

**Published:** 2022-05-17

**Authors:** Sileshi Tadesse Gemeda, Zinash Beyene Woldemariam

**Affiliations:** 1 Department of Pharmacology, Yekatit 12 Hospital Medical College, Addis Ababa, Ethiopia; 2 Department of Pharmacy, Rift Valley University, Adama, Ethiopia

**Keywords:** Diabetes Mellitus, Self-management Practice, Associated Factors

## Abstract

**Introduction:**

There is almost no published data on the assessment of self-management practice among adult type II diabetes patients in Ethiopia. Hence, we aim to assess the level of self-management practice for people with type II diabetes patients attending Adama Hospital Medical College, Ethiopia.

**Method:**

The study was conducted from April 1 to August 30, 2021 in type II diabetes mellitus patients at Adama Hospital Medical College. The survey was performed using the diabetes mellitus self-Management questionnaire, which consists of four domains Physical activity, Physician contact, Medication adherence, glucose management and dietary management of the patients. The data was analyzed using Statistical Package for Social Science (SPSS) version 20.0. Descriptive statistics was performed. Fisher's Exact Test was used to determine the presence of association between adherence to self-care behavior and other variables. P-value less than 0.05 determines statistical significance.

**Result:**

Majority (63.4%) of respondents do not perform self-monitoring of blood glucose (SMBG). Out of a total of 93 participants, 48 (51.6%) respondents adhered to insulin therapy. Sixty-two (66.7%) adhered to recommended diet management practice, 57 (61.3%) did not adhere to physical activity recommendations and 59 (63.5%) participants adhered to overall self-care practice (DMSQ).

**Conclusion:**

Although the importance of self-care practices in the management of diabetes were recognized to be useful and effective for achieving glycemic control and preventing serious diabetes complications, our study found that most patients had not adhered to self-care practice especially in terms of SMBG and physical activity. Overall (DSMQ) adherence to self-care practice was optimal amongst type II diabetes patients in AHMC Chronic care unit.

## Introduction

Diabetes mellitus (DM) is defined as a metabolic disorder of multiple etiologies characterized by chronic hyperglycemia with disturbance in carbohydrate, protein and fat metabolism resulting from defect in insulin secretion, insulin action or both. The World Health Organization has projected that in 2030 the global prevalence of DM in all age ranges wild becomes 4.4% or a total of 366 million diabetes patients. The Ethiopian diabetes association (EDA) estimated 2–3% prevalence in 2013 in Ethiopia [1–3]. Diabetes reduces both quality of life and life expectancy and imposes large economic burdens on individuals and on national health care systems directly or indirectly [4].

Acute and Long-term complications of diabetes can be reduced by following ongoing patient self-managements. Effective diabetes self-care can be achieved using the following actions: lifestyle modification, diet control, regular physical exercise, smoking cessation, weight reduction, self-blood glucose monitoring, medication adherence and foot care are effective treatments for improving diabetes consequences; so that self-care behavior adherence and patient education are the first steps in helping patients to better care and manage their disease [3–7]. About 95% of diabetes treatment depends on self-care behaviors regardless of the type of diabetes and similarly the patients and their families accomplish the self-care, hence diabetes patients must correct their behavior like adherence to prescribed medication, diet control, and changes to physical activity especially for older patients to prevent diabetes complications, which may be potentially lethal [8, 9]. Therefore, this study was aimed to identify personal perceptions of diabetes patients towards self-care behaviors.

Different studies done in different countries showed that Self-reported adherence to medication was low. A cross sectional study was done on physical activity and reported barriers to activity among type II diabetes patients in United Arab Emirates, Jimma University and Arba Minch (Ethiopia) and the result of the study showed that of the 390 patients recruited, only 25% reported an increase in their physical activity levels following the diagnosis of diabetes and only 3% reported physical activity levels that meet the recommended guidelines [10–18]. Since there is no research on diabetes self-care management practice in Adama, we hope that our study will provide better solutions for the patients, the health professionals, and the governmental and non-governmental health facilities.

## Methods

### Study area and sampling

The study was conducted from April 1 to August 30, 2021 in Adama Hospital medical college located in Adama Town. Adama is located at 99 km away from the capital city of Ethiopia, Addis Ababa, to the east. According to the central statistical agency report in 2007 E.C, the town has a total population of 341,796 (male 170,838 and female 170,953). The information from Adama town health Bureau shows that currently the town has 1 governmental Medical college hospital, 7 government health centers, 4 private Hospital, 94 private clinics with different level of which 17 special privet clinics, 4 Non-Governmental clinics and 108 drug store and Pharmacies. The college hospital, AHMC, has catchment population of about 5.2 million serving as referral hospital for all nearby district hospitals and the adjacent regions. Currently the hospital provides a service for 106 type II Diabetes patients. A cross-sectional study design was used and data was collected by using semi-structured questionnaire through face to face interview. Source populations were all type II diabetes who visited AHMC for diabetes follow-up care. Study subjects were those aged, 18 years and older diagnosed with type II diabetes who visited the hospital at the time of data collection period and fulfills eligibility criteria. Before starting the data collection, questionnaires were prepared in English and translated into Amharic and translated back into English to check its consistency. The Amharic versions were used for data collection after pretesting on 5% (9 patients) of the actual sample size in AHMC type II diabetes. One clinical pharmacy, one nurse for data collection and one medical doctor (MD) working in the hospital for supervision were given orientation before data collection. During the study period the effect of different factors affecting self-glucose management practice, dietary control practice, medication adherence practice, physical exercise practice, physician contact practice, and overall practice were assessed. Continuous follow up and supervision was made by the principal investigators throughout the data collection period. The data was analyzed by Statistical Package for Social Science (SPSS) version 20.0. Descriptive statistics was used for most variables; a Fischer Exact Analysis was employed to determine the presence of association between adherences to self-care behavior with other variables at P-value less than 0.05.

### Sample size determination

Sample size was determined by using single population proportion.

*n* = (Z1-α/2)^2^ p (1–p)/d^2^ = (1.96)^2^*0.49(1–0.49)/ (0.05)^2^ = 384, Where: *n* = the required sample size.

Z = the standard normal deviation, set at 1.96 (for 95% confidence interval), *P* = prevalence of poor self-care behaviors toward diabetes = 49%, d = the margin of error (precision) = 5%. The final sample size was determined as follows by using the following correction / reduction formula for the estimated Sample size < 10,000 as a following: nf = ni/(1 + ni/N), Where: nf = The final sample size, ni = Initial sample size 384, *N* = Estimated total sample size of T2DM patient = 106, nf = 384/ (1 + 384/106) = 83.08, Considering a 10% non-response rate, the total sample size was:

10/100*83 = 8.3 + 83 = 91.3. So, by adding non-response rate to the final simple size it gives 91.3. Hence, 93 Type II diabetes patients were included in this study.

### Eligibility criteria

Patients who were 18 years of age or older and diagnosed with type II diabetes, residents of the catchment area, neither mentally nor physically handicapped, and not having acute illness as opposed to chronic disease like DM.

### Ethical consideration

Ethical clearance was obtained from Institutional Review Board (IRB) of Rift Valley University for research to be performed in accordance with the Declaration of Helsinki. Interview was carried out only with full informed consent of the patient being interviewed. Each patient was assured that the information provided by him/ her was confidential and used only for the purpose of research. Patients were allowed to refuse or discontinue participation at any time they want.

## Results

### Socio demographic characteristics

A total of 93 male and female adult type II diabetes patients were interviewed using prospective cross-sectional standardized structure questionnaire and included in the analysis. Of all patients 52 (55.9%) and 41 (41.1%) were female and male respectively. The majority of the study participants were in the age group of 38 to 67 years. The mean age in which diabetes disease started was 52.8 ± 14.7 years with minimum age of 24 and maximum age of 81. The mean duration of diabetes was 11.11 ± 8.019 years with minimum of 1 year and maximum of 34 years. The BMI of the respondents was 23.2 + 3.2 with minimum BMI of 16 and maximum BMI of 34.7 and FBS of the patients was by mean of 177.88 + 49.24 with minimum of 71 and maximum of 358. Sixty-one (65.6%) were married, 30 (32.3%) completed primary level of education and 34 (36.6%) were housewives (Table 1).

**Table 1:** Patients sociodemographic characteristics (*n* = 93)

Characteristics	Mean ± SD/ Frequency
Age (Year)	52.83 ± 14.7
BMI (Kg/m2)	23.1 ± 3.2
Duration of Diabetes (Year)	11.11 ± 8.0
FBS Mean (mg/dl)	177.9 ± 49.2
Gender	
Male Female	41(44.1) 52(55.9)
Education	
Can't read and write Can read and write Primary school (1–8) Secondary school (9–12) Collage/university	16(17.2) 12(12.9) 30(32.3) 17(18.3) 18(19.4)
Marital status	
Single Married Divorced Widowed/er	12(12.9) 61(65.6) 4(4.3) 16(17.2)
Employment/occupation	
Employed Unemployed	18(19.4) 6(6.5)
Merchant Farmer House wife Retired Daily laborer Others	9(9.7) 4(4.3) 34(36.6) 12(12.9) 6(6.5) 4(4.3)

*FBS* Fasting Blood Sugar, *SD* standard deviation

### Knowledge and attitude towards diabetes

The majority of the patients, 63 (67.7%), had negative attitude towards the disease, thus illustrating that most of the patients were pessimistic towards diabetes. Fifty-four (58.1%), subjects had good knowledge about diabetes and its care principles. Overall, patients had good knowledge on diabetes and its self-care practices when asked about causes, types and management principles of diabetes, accordingly (Table 2).

**Table 2:** Knowledge and Attitude about Adults with Type II diabetes in AHMC (*n* = 93)

Attitude and Knowledge towards diabetes	Attitude	Knowledge
Positive (%)	30 (32.3)	54(58.1)
Negative (%)	63(67.7)	39(41.9)

### Co-morbidity conditions and treatment intensity

Twenty-two (23.7%) have Hypertension and 2 (2.2%) Ischaemic heart disease. The other medical conditions reported are Asthma and Chronic Kidney Disease (CKD), and chronic complications are Peripheral Neuropathy, and Retinopathy. From the total of study participants about 48 (51.6%) followed by 41(44.1%) and 4 (4.3%) had utilized insulin therapy, oral hypoglycemic agents (OHA), and both (OHA & Insulin) respectively (Table 3).

**Table 3:** Type of comorbidities and treatment type among Type II adult diabetes patients

Type of comorbidities and treatment type		Number (%)
Type of comorbidities	HTN	22 (23.7)
	IHD	2 (2.2)
	Dyslipidemia	8 (8.6)
	Other	14 (15)
	None	47 (50.5)
Type of treatment	Oral hypoglycemic agent	41 (44.1)
	Insulin	48 (51.6)
	Both	4 (4.3)

### Self-care practice domains and adherence conditions

From the different self-practice domains patients showed greater adherence towards medication adherence (97.8%), health care (86%), dietary plan (66.7%), DSMQ (63.5%) and poor adherence SMBG (38.7%), and physical activity (32.3%). Majority of participants (65.5%) were not adhered to SMBG which means, monitored less than 7 times per week and almost all patients alleged that they did SMBG practices when they had symptoms of hyperglycemia or hypoglycemia. Presence of glucometer at home, education and monthly income was found to have statistically significant association with adherence to SMBG practice (Table 4).

**Table 4:** Adherence level self-care practice (*n* = 93)

Type of self-care practice	level of adherence	
	Adherent (%)	Non-adherent (%)
SMBG	30 (34.4)	63 (65.5)
Medication	91 (97.8)	2 (2.2)
Health care	80 (86)	13 (14)
Dietary plan	60 (64.5)	33 (35.5)
Physical activity	30 (32.3)	63 (67.7)
DSMQ status	60 (64.5)	33 (35.5)

Among factors listed, presence of co-morbidities (*p* = 001) and educational status (*p* = 0.037) have significant association with self-glucose management practices. The other factors like consumption of social drugs, glucometer presence at home, diet management and presence of complications don’t have significant association (Table 5).

**Table 5:** Associated factors of DMSQ glucose management practices in patients with type II diabetes at AHMC (*n* = 93)

DMSQ Glucose Management practice			
Variables	Poor	Optimal	*P* value
Presence of comorbidities			
Yes No	32(72.7%) 29(59.2%)	12(27.3%) 20(40.8%)	0.001
Family History			
Yes No	32(72.7%) 29(59.2%)	12(27.3%) 20(40.8%)	0.195
Attitude towards Diabetes			
Negative Positive	45(71.4%) 16(53.3%)	18(28.6%) 14(46.7%)	0.105
Social drugs use			
Khat Alcohol Two from the 3 All None	2(100%) 11(84.6%) 2(100%) 2(100%) 44(59.5%)	0(0%) 2(15.4%) 0(0%) 0(0%) 30(40.5%)	0.184
Education			
Can’t read and write Can read and write Primary school (1–8) Secondary school (9–12) College/university	10(62.5%) 8(66.6%%) 14(46.7%) 13(76.5%) 16(88.8%)	6(37.5%) 4(33.3%) 16(53.3%) 4(23.5%) 2(11.2%)	0.037
Glucometer at home			
No Yes	43(64.2%) 18(69.2%)	24(35.8%) 8(30.8%)	0.809
Diet management			
No Yes	27(72.9%) 34(60.7%)	10(27.0) 22(39.3%)	0.269
Presence of complications			
No Yes	21(53.8%) 40(74.1%)	18(46.2%) 14(25.9%)	0.05

A Fischer Exact test of independence was performed to the relationship between different factors and medication adherence. Among the factors physical exercise and marital status has significant association (*p* = 0.007) and (*p* = 001) respectively. The other factors like family history of diabetes, patients attitude, presence of glucometer at home etc. didn’t have a significant association (Table 6).

**Table 6:** Associated factors of DMSQ medication adherence practices in patients with type II diabetes at AHMC (*n* = 93)

Medication Adherance				
Variables		poor	optimum	*P*-value
Hospitalization due to diabetes	No Yes	2(3.2%) 0(0%)	61(96.3%) 30(100%)	1.00
Patients attitude	Positive Negative	0(0%) 2(3.2%)	30(100%) 61(96.3%)	0.558
Family history of diabetes	Yes No	2(4.5%) 0(0%)	42(95.5%) 49(100%)	0.221
Presence of glucometer at home	Yes No	0(0%) 2(3%)	26(100%) 65(97.0%)	1.00
Physical exercise	Walking Gymnastic Other None	0(0%) 2(33.3%) 0(0%) 0(0%)	66(100%) 4(66.7%) 15(100%) 6(100%)	0.007
Experience of hypoglycemia	Experienced Not-experienced	0(0%) 2(3.6%)	38(100%) 53(96.4%)	0.511
Diet control	No yes	2(5.4%) 0(0%)	35(94.6%) 56(100%)	0.156
Diabetes follow up	Always Sometimes	0(0%) 2(6.5%)	62(100%) 29(93.6%)	0.109
Diabetes complication	No Yes	2(5.1%) 0(0%)	37(94.9%) 54(100%)	0.173
Presence of comorbidities	Hypertension IHD Dyslipidemia Other none	0(0%) 0(0%) 0(0%) 0(0%) 2(4.3%)	22(100%) 2(100%) 8(100%) 14(100%) 45(95.8%)	1.00
Marital status	Single married divorced widowed/er	0(0%) 0(0%) 2(50%) 0(0%)	12(100%) 61(100%) 2(50%) 16(100%)	0.001
Sex of patients	Male female	0(0%) 2(3.8%)	41(100%) 50(96.2%)	0.502

Presence of comorbidities (*p* = 0.024), diabetic complications (*p* = 0.08) and physical exercise (*p* = 0.012) significantly affected self-care dietary management practices as compared other factors listed in the table below (Table 7).

**Table 7:** Associated factors of DMSQ dietary management practices in patients with type II diabetes at AHMC (*n* = 93)

Dietery management status				
Variables		poor	Optimal	*P*-value
Sex of patients	Male Female	11(26.8%) 20(38.5%)	30(73.2%) 32(61.5%)	0.273
Marital status	Single Married Divorced Widowed/er	2(16.6%) 25(40.9%) 2(50%) 2(12.5%)	10(83.3%) 36(59.1%) 2(50%) 14(87.5%)	0.068
Social drug that the patient consumes	Khat Alcohol None Two from the three All	2(100%) 5(38.5%) 24(32.4%) 0(0%) 0(0%)	0(0%) 8(61.5%) 50(67.6%) 2(100%) 2(100%)	0.271
Presence of co morbidities	Hypertension IHD Dyslipidemia Other None	10(45.5%) 2(100%) 0(0%) 6(42.8%) 13(27.6%)	12(54.5%) 0(0%) 8(100%) 8(57.1%) 34(72.3%)	0.024
Diabetes complication	No Yes	7(17.9%) 24(44.4%)	32(82.1%) 30(55.6%)	0.08
Diabetes follow up	Always Sometimes	18(29.0%) 13(41.9%)	44(70.9%) 18(58.1%)	0.248
Dietary plan the patient set with doctor	No Yes	17(45.9%) 14(25%)	20(54.1%) 42(75%)	0.045
Experience of hypoglycemia	Experienced Not-experienced	16(42.1%) 15(27.3%)	22(57.9%) 40(72.7%)	0.180
Physical exercise	Walking Gymnastic Other None	22(33.3%) 0(0%) 9(60%) 0(0%)	44(66.7%) 6(100%) 6(40%) 6(100%)	0.012

As can be seen in Table 8, with an exception of diabetes follow up (*p* = 0.012) the other factors don’t have significant association with physical exercise practice.

**Table 8:** Associated factors of DMSQ physical exercise practices in patients with type II diabetes at AHMC (*n* = 93)

Physical exercise practice				
Variable		Poor	Optimum	*P*-value
Sex of patients	Male	25(60.9%)	16(39.0%)	1.00
	Female	32(61.5%)	20(38.5%)	
Marital status	Single	6(50%)	6(50%)	0.57
	Married	37(60.65%)	24(39.4%)	
	Divorced	2(50%)	2(50%)	
	Widowed/er	12(75%)	4(25%)	
Presence of co-morbidities	Hypertension	12(54.5%)	10(45.5%)	0.052
	IHD	2(100%)	0	
	Dyslipidemia	8(100%)	0	
	Other	6(42.8%)	8(57.2%)	
	None	29(61.7%)	18(38.3%)	
Diabetes complication	No	21(53.8%)	18(46.3%)	0.281
	Yes	36(66.6%)	18(33.4%)	
Diabetes follow up	Always	44(70.9%)	18(29.1%)	0.012
	Sometimes	13(41.9%)	18(58.1%)	
Dietary management	No	23(62.2%)	14(37.8%)	1.00
	Yes	34(60.7%)	22(39.3%)	
Experience of hypoglycemia	Experienced	26(68.4%)	12(31.6%)	0.283
	Not experienced	31(56.4%)	24(43.7%)	
Physical exercise	Walking	42(63.6%)	24(36.4%)	0.002
	Gymnastic	0	6(50%)	
	Other	9(60%)	6(40%)	
	None	6(100%)	0	
Glucometer at home	Yes	12(46.2%)	14(53.9%)	0.096
	No	45(67.2%)	22(32.8%)	
Family history	Yes	26(59.1%)	18(40.9%)	0.831
	No	31(63.3%)	18(36.7%)	
Patients attitude	Positive	16(53.3%)	14(46.7%)	0.363
	Negative	41(65.1%)	22(34.9%)	
Hospitalized due to diabetes	No	37(58.7%)	26(41.3%)	0.503
	Yes	20(66.6%)	10(33.4%)	

Among all factor’s, marital status (*p* = 0.024) and diabetes follow up (*p* = 0.002) have greatly affected the self-care physician contact practices of the patients (Table 9).

**Table 9:** Associated factors of DMSQ physician contact practices in patients with type II diabetes at AHMC (*n* = 93)

Physician contact status				
Variable		Poor	Optimum	*P*-value
Sex of patients	Male	4(9.8%)	37(90.2%)	0.540
	Female	8(15.4%)	44(84.6%)	
Marital status	Single	0(0%)	12(100%)	0.024
	Married	10(16.4%)	51(83.6%)	
	Divorced	2(50%)	2(50%)	
	Widowed/er	0(0%)	16(100%)	
Social drug use	Khat	0(0%)	2(100%)	0.735
	Alcohol	3(23.1%)	10(76.9%)	
	None	9(12.2%)	65(87.8%)	
	Two from the three	0(0%)	2(100%)	
	All	0(0%)	2(100%)	
Experience of hypoglycemia	Experienced	3(7.9%)	35(92.1%)	0.348
	Not experienced	9(16.4%)	46(83.6%)	
Dietary plan	No	7(18.9%)	30(81.1%)	0.210
	Yes	5(8.9%)	51(91.1%)	
Presence of co morbidities	Hypertension	0(0%)	22(100%)	0.086
	IHD	0(0%)	2(100%)	
	Dyslipidemia	0(0%)	8(100%)	
	Other	2(14.3%)	12(85.7%)	
	None	10(21.3%)	37(78.7%)	
Glucometer at home	Yes	3(11.5%)	23(88.5%)	1.00
	No	9(13.4%)	58(86.6%)	
level of education	Can't read and write	2(12.5%)	14(87.5%)	0.269
	Can read and write	2(16.67%)	10(83.3%)	
	Primary school (1–8)	2(6.7%)	28(93.3%)	
	Secondary school (9–12)	1(5.9%)	16(94.1%)	
	Collage/university	5(27.9%)	13(72.2%)	
Diabetes follow up	Always	3(4.8%)	59(95.2%)	0.002
	Sometimes	9(29.0%)	22(70.9%)	

It is observed that the overall self-care practices of the patients were significantly affected by the sex of the patients (*p* = 0.03), marital status (*p* = 0.035), and presence of comorbidities (*p* = 0.021) (Table 10).

**Table 10:** Associated factors of DMSQ overall self-care practices in patients with type II diabetes at AHMC (*n* = 93)

DMSQ over all status				
Variable		Poor	Optimum	*P*-value
Sex of patients	Male	8(19.5%)	33(80.5)	0.03
	Female	26(50%)	26(50%	
Marital status	Single	2(16.7%)	10(83.3%	0.035
	Married	22(36.1%)	39(63.9%)	
	Divorced	4(100%)	0(0%)	
	Widowed/er	6(37.5%)	10(62.5%	
Social drug that the patient consumes	Khat	0(0%)	2(100%)	0.133
	Alcohol	8(61.5%)	5(38.5%	
	None	26(35.1%)	48(64.9%)	
	Two from the three	0(%)	2(100%)	
	All	0(0%)	2(100%)	
Experience of hypoglycemia	Experienced	16(42.1%)	22(57.9%)	0.388
	Not experienced	18(32.7%)	37(67.3%)	
Dietary plan	No	14(37.8%)	23(62.2%)	1.00
	Yes	20(35.7%)	36(64.3%)	
Presence of co morbidities	Hypertension	12(54.5%)	10(45.5%	0.021
	IHD	2(100%)	0(0%)	
	Dyslipidemia	4(50%)	4(50%)	
	Other	2(14.3%)	12(85.3%)	
	None	14(30%)	33(70%)	

## Discussion

Inadequate diabetes self-management remains a significant problem facing health care providers and populations in all settings. Patients, who have adequate self-management have better outcomes, live longer, enjoy a higher quality of life, and suffer fewer symptoms and minimal complications. Diabetes self-management strategies increase lifestyle adjustments to maintain best possible diabetes management to achieve optimal glycemic control in patients with type II diabetes [4, 5]. In this study the current situation of adherence to self-care practices of patients with type II diabetes in AHMC and factors that contribute to efficient adherence to self-care practices of diabetes was investigated. Accordingly, about 48(51.6%) practiced the recommended self-care practices activities which is similar with the study done Jimma University specialized Hospital (50.1%) [17].

High level of diabetic knowledge was the reference group practice which is better than study conducted in Nekemte Referral Hospital (45%). This variation could be due to difference in glycemic target range used, instruments used to self-care practices adherence. From the total of study participants, the treatment intensity of the patients was insulin therapy 48(51.6%), oral hypoglycemic agents 41(44.09%) and both treatment 4(4.3%) respectively when compared to the treatment intensity of the patients done in Black Lion Specialized Hospital, Ethiopia [6, 7, 19].

This study also showed that only 34.4% were adhered to SMBG (self-monitoring of blood glucose) practices. This result is slightly lower than study done in Ethiopia (36.1%) elsewhere. Although SMBG is recognized to be useful and effective in achieving diabetes control, this study has found that only a minority of patients with diabetes were performed SMBG practices. This is probably related to lack of awareness on its importance in the management of diabetes and there are relevant financial barriers to purchase the device and its strips [18].

There was no significant association between education, Glucometer at home, diet control knowledge and presence of complications, presence of co morbidities and self-care glucose management practices. When compared with the study done in TASH Majority of participants (63.9%) were not adhered to self-monitoring of blood glucose which means, even almost all patients were said that they did SMBG practices when they had symptoms of hyperglycemia or hypoglycemia or at the time of health care visit and so that only (36.1%) were adhered which means monitored at least highly estimated monthly income were adhered two times more than counterpart which is similar with our study [20].

According to this study those patients with comorbid conditions, with family history of diabetes, who did not follow a physical exercise were more likely to exercise poor self-care medication adherence than patients with the listed conditions. Patients who were married, single, widow/er, who were not having glucometer at home, who didn’t follow diet control, who didn’t experience hypoglycemic episode, were more likely to exercise good self-care medication adherence practice. Patients who were hospitalized due to diabetes were more likely to exercise optimal self-care medication adherence practice when compared to patients who were not hospitalized due to diabetes and this study is in line with the study done in Cambodia [21].

There was no significant association between genders and diabetes follow up and self-care medication adherence practices. When compared with the study done in TASH total of (63%) study participants were adhered with prescribed anti-diabetes drugs but out of the total study subjects (37%) were not adhered. Of the whole adhered patients (60.3%) and (66%) were female and male respectively. In other hand the treatment intensity of the patients were oral hypoglycemic agents (28.4%), insulin therapy (59.6%) and both treatment (12%). Individuals who took insulin injection as treatment intensity were three times adhered than those who took oral hypoglycemic agents. However, no association to other health status data and socio demographic characteristics which is similar with our study by adherence and type of treatment highly taken [7, 20].

Patients with comorbid conditions were more likely to exercise poor self-care dietary management practice than were patients who did not had co-morbid conditions. Patients who did not follow a physical exercise plan were more likely to exercise poor self-care dietary management practice when compared to patients who followed a physical exercise plan. Patients who didn’t preferred special diet were more likely to exercise poor self-care dietary management practice when compared to patients who preferred special diet. Female patients were more likely to exercise poor self-care dietary management practice when compared to male patients. Patients who abused social drugs were more likely to exercise poor self-care dietary management practice when compared to male patients, there was no significant association between presence of complications, knowledge of complications, diabetes follow up, experienced hypoglycemia, marital status and self-care dietary management practice.

Majority (58.6%) of the study participants were not adhered to recommended diet management practices and only (41.4%) of patients were adhered which means follow recommended diet management practices as compared to the study conducted in TASH. This study showed contrast result in TASH where participants with high level of education were about three times more likely to be adhered to diet management practices when compared with their counterparts and patients with high monthly income were showed six times more engaged in diet management practices when compared with very low monthly [20].

Patients who preferred diet control were more likely to exercise good self-care physical management practice when compared to patients who did not prefer diet control. Patients who did not follow a physical exercise plan were more likely to exercise poor self-care physical management practice when compared to patients who followed a physical exercise plan. One possible reason for the diverge results of the present study may be due to the behavior of patients regarding medication adherence. Some diabetes drugs cause a hypoglycemic complication, so patient who adhere the medication can face hypoglycemic episode.

Patients with family history of diabetes were more likely to exercise optimal self-care physical management practice than were patients with no family history of diabetes. On the other hand, findings of a study conducted in Nepal indicated that only 84 (21.4%) of the patients with family history of diabetes adhere to physical activity and 79.1% were not adhered which is lower than our study [21].

There was no significant association between education, follow-up, gender and self-care physical management practice. When compared with the study done in TASH of the total study participants one hundred fifty-nine (49.1%) were reported adhered to physical activities that exactly meet the recommended guidelines and about one hundred sixty-fives were not adhered. Single marital status participants and who attended higher level of education showed a significant association to their adherence condition which is about five times and two times with regard to physical activity relatively when compared with their counterparts and in the same way also patients who had greater average monthly income were adhered to the physical activity practices about three times which is similar result with our study by adherence which is slightly poor [20]. When compared with the study done in Arba Minch of the total study participants 169 (87.1%) of patients had good relationship with professionals caring for their diabetes and only 13(6.7%) respondents had poor relationship with health professionals caring for their diabetes [22].

Patients who were single were more likely to exercise good self-care overall management practice when compared to patients who were married, divorced and widow/er. Male patients were more likely to exercise self-care overall management practice than female patients. Those patients who had hypoglycemic episode, who didn’t prefer special diet were less likely to exercise optimal self-care overall management as compared to patients who didn’t experienced hypoglycemic and who preferred special diet. There was no significant association between presence of co morbidities, social drugs abuse and overall self-care management practice. When compared with the study done in TASH Self-care practice were reported adhered in (51.5%) patients and not adhered in (48.5%) participants. There was statistically significant association between females with higher level of education and adherence level to overall diabetes self-care practice and about two and three times more likely to be engaged in self-care practices when compared with male and illiterate participants. Similarly, those participants who have high income were adhered two times more which is similar result with our study [20].

## Conclusion

The important role of self-care practices in management of diabetes were recognized to be useful and effective in achieving diabetes control and preventing its serious complications. However, this study revealed that; adherence to self-care practices particularly physical activity, self-monitoring of blood glucose and glycemic control of adult type II diabetes was low. In this study patients had high level of knowledge and attitude on diabetes. The patients gender, marital status and hypoglycemic episode experience are independent associated factors for adherence to overall self-care practices towards type II DM.

## Data Availability

The datasets generated and/or analyzed during the current study are not publicly available due fear of data using by another person but are available from the corresponding author on reasonable request.

## Abbreviations

ADA: American diabetes association
AHMC: Adama Hospital Medical College
BMI: Body mass index
DKT: Diabetic Knowledge Test
DM: Diabetes mellitus
DSMQ: Diabetes self-management questionnaires
EC: Ethiopian calendar
EDA: Ethiopian diabetes association
FBG: Fasting blood glucose
FBS: Fasting blood sugar
IDF: International diabetes federation
IRB: Institutional review board
MMAS: Morisky medication adherence scale
OHA: Oral hypoglycemic agent
RVU: Rift Valley University
SDSCA: Summary of diabetes self-care Activities
SMBG: Self–monitoring blood glucose
SPSS: Statistical packaging for social sciences
TASH: Tikur Anbessa Specialized Hospital
T2DM: Type II diabetes mellitus
WHO: World Health Organization
